# Virus taxonomy and the role of the International Committee on Taxonomy of Viruses (ICTV)

**DOI:** 10.1099/jgv.0.001840

**Published:** 2023-05-04

**Authors:** Stuart G. Siddell, Donald B. Smith, Evelien Adriaenssens, Poliane Alfenas-Zerbini, Bas E. Dutilh, Maria Laura Garcia, Sandra Junglen, Mart Krupovic, Jens H. Kuhn, Amy J. Lambert, Elliot J. Lefkowitz, Małgorzata Łobocka, Arcady R. Mushegian, Hanna M. Oksanen, David L. Robertson, Luisa Rubino, Sead Sabanadzovic, Peter Simmonds, Nobuhiro Suzuki, Koenraad Van Doorslaer, Anne-Mieke Vandamme, Arvind Varsani, F. Murilo Zerbini

**Affiliations:** ^1^​ School of Cellular and Molecular Medicine, Faculty of Life Sciences, University of Bristol, University of Bristol, Bristol, UK; ^2^​ Nuffield Department of Medicine, University of Oxford, Oxford, UK; ^3^​ Quadram Institute Bioscience, Norwich Research Park, Norwich, UK; ^4^​ Departamento de Microbiologia/BIOAGRO, Universidade Federal de Viçosa, Viçosa, MG, Brazil; ^5^​ Institute of Biodiversity, Faculty of Biological Sciences, Cluster of Excellence Balance of the Microverse, Friedrich-Schiller-University Jena, Jena, Germany; ^6^​ Theoretical Biology and Bioinformatics, Science for Life, Utrecht University, Utrecht, The Netherlands; ^7^​ Instituto de Biotecnología y Biología Molecular, CCT-La Plata, CONICET, UNLP, La Plata, Buenos Aires, Argentina; ^8^​ Institute of Virology, Charité-Universitätsmedizin Berlin, Corporate Member of Free University Berlin, Humboldt-University Berlin, and Berlin Institute of Health, Berlin, Germany; ^9^​ Institut Pasteur, Université Paris Cité, CNRS UMR6047, Archaeal Virology Unit, Paris, France; ^10^​ Integrated Research Facility at Fort Detrick (IRF-Frederick), National Institute of Allergy and Infectious Diseases, National Institutes of Health, Fort Detrick, Frederick, Maryland, USA; ^11^​ Division of Vector-Borne Diseases, National Center for Emerging and Zoonotic Infectious Diseases Centers for Disease Control and Prevention, Fort Collins, Colorado, USA; ^12^​ Department of Microbiology, University of Alabama at Birmingham (UAB), Birmingham, Alabama, USA; ^13^​ Institute of Biochemistry and Biophysics of the Polish Academy of Sciences, Warsaw, Poland; ^14^​ Division of Molecular and Cellular Biosciences, National Science Foundation, Alexandria, Virginia, USA; ^15^​ Molecular and Integrative Biosciences Research Programme, Faculty of Biological and Environmental Sciences, University of Helsinki, Helsinki, Finland; ^16^​ MRC-University of Glasgow Centre for Virus Research, Glasgow, UK; ^17^​ Istituto per la Protezione Sostenibile delle Piante, CNR, SS Bari, Bari, Italy; ^18^​ Department of Biochemistry, Molecular Biology, Entomology and Plant Pathology, Mississippi State University, Mississippi, USA; ^19^​ Institute of Plant Science and Resources, Okayama University, Kurashiki, Okayama, Japan; ^20^​ School of Animal and Comparative Biomedical Sciences, Department of Immunobiology, BIO5 Institute, Genetics Graduate Interdisciplinary Program, Cancer Biology Graduate Interdisciplinary Program and University of Arizona Cancer Center, Tucson, Arizona, USA; ^21^​ KU Leuven, Department of Microbiology, Immunology and Transplantation, Rega Institute for Medical Research, Clinical and Epidemiological Virology, Leuven, Belgium and Center for Global Health and Tropical Medicine, Instituto de Higiene e Medicina Tropical, Universidade Nova de Lisboa, Lisbon, Portugal; ^22^​ The Biodesign Center for Fundamental and Applied Microbiomics, School of Life Sciences, Center for Evolution and Medicine, Arizona State University, Tempe, Arizona, USA; ^23^​ Departamento de Fitopatologia/BIOAGRO, Universidade Federal de Viçosa, Viçosa, MG, Brazil

**Keywords:** virus taxonomy, virus classification, virus nomenclature, International Committee on Taxonomy of Viruses, ICTV

## Abstract

The taxonomy of viruses is developed and overseen by the International Committee on Taxonomy of Viruses (ICTV), which scrutinizes, approves and ratifies taxonomic proposals, and maintains a list of virus taxa with approved names (https://ictv.global). The ICTV has approximately 180 members who vote by simple majority. Taxon-specific Study Groups established by the ICTV have a combined membership of over 600 scientists from the wider virology community; they provide comprehensive expertise across the range of known viruses and are major contributors to the creation and evaluation of taxonomic proposals. Proposals can be submitted by anyone and will be considered by the ICTV irrespective of Study Group support. Thus, virus taxonomy is developed from within the virology community and realized by a democratic decision-making process. The ICTV upholds the distinction between a virus or replicating genetic element as a physical entity and the taxon category to which it is assigned. This is reflected by the nomenclature of the virus species taxon, which is now mandated by the ICTV to be in a binomial format (genus + species epithet) and is typographically distinct from the names of viruses. Classification of viruses below the rank of species (such as, genotypes or strains) is not within the remit of the ICTV. This article, authored by the ICTV Executive Committee, explains the principles of virus taxonomy and the organization, function, processes and resources of the ICTV, with the aim of encouraging greater understanding and interaction among the wider virology community.

## ICTV organization

First established in 1966 as the International Committee on Nomenclature of Viruses (ICNV) and changing its name to the International Committee on Taxonomy of Viruses (ICTV) in 1975, the ICTV oversees the classification and taxonomic nomenclature of viruses and some other mobile genetic elements, including satellite nucleic acids, viriforms and viroids [1]. The ICTV is a committee of the Virology Division of the International Union of Microbiological Societies (IUMS), and ICTV-authorized classification and nomenclature serve as the official reference point for virus taxonomy (see Glossary for definitions). There is a distinction between the ICTV taxonomy and other ways of classifying viruses, such as the Baltimore classification [2, 3] or the grouping of viruses into non-phylogenetic assemblages (such as “arboviruses” or “mycoviruses”) based on phenotypic characteristics. Thus, while there can be multiple classification systems, there is only one approved virus taxonomy [4]. ICTV affairs are governed by its Statutes (https://ictv.global/statutes), and the rules of virus taxonomy are explained in the International Code of Virus Classification and Nomenclature (ICVCN) (https://ictv.global/code).

**Fig. 1.**
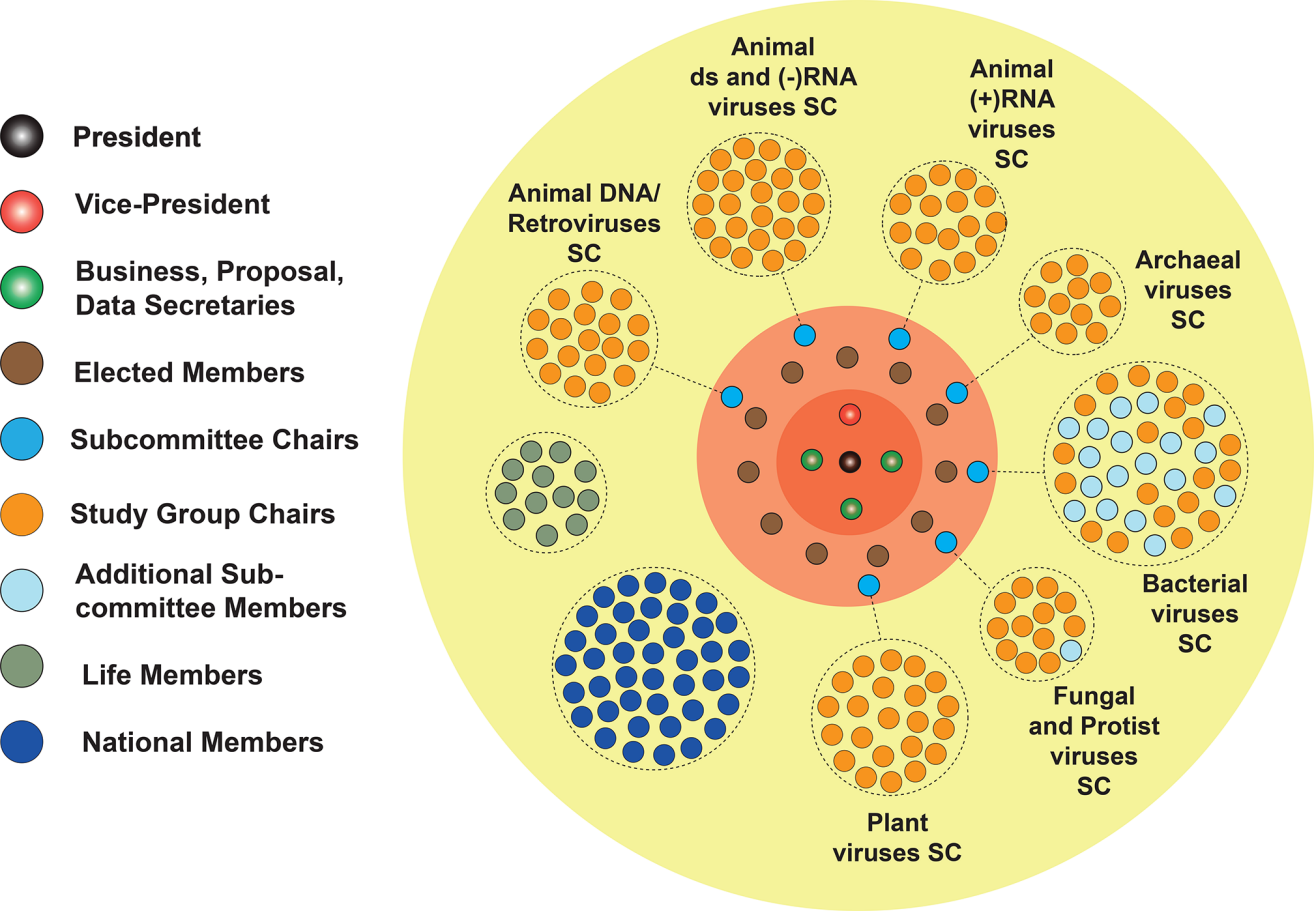
The organization of the ICTV. ICTV members (yellow circle) include the Officers (dark orange circle), Elected Members including Subcommittee chairs (light orange circle), Life Members, National Members and Study Group chairs. The Bacterial Viruses Subcommittee and Fungal and Protist Virus Subcommittee also include ICTV members who are not Study Group chairs. The Officers, Elected Members, and Life Members are elected by a vote of the full ICTV membership. National Members are nominated by Member Societies of the Virology Division of the International Union of Microbiological Societies (IUMS). Members of Virus Subcommittees are appointed by the Virus Subcommittee chairs (who are themselves appointed from the Elected members by the Executive Committee) and include all Study Group chairs. An unlimited number of Study Group members are assembled by the Study Group chairs for their expertise on viruses of a particular taxon but are not part of the ICTV. The names of the many hundreds of Study Group Members are published on the ICTV website to recognize their valuable contribution to virus taxonomy (https://ictv.global/sc/dna, https://ictv.global/sc/rna-m, https://ictv.global/sc/rnas, https://ictv.global/sc/archaeal, https://ictv.global/sc/bacterial, https://ictv.global/sc/fungal-protist, https://ictv.global/sc/plants).

The ICTV comprises an Executive Committee (EC) of 23 members, about 40 National Members (representing virologists in different countries), 11 Life Members, the chairs of over 120 taxon-specific Study Groups, and additional Subcommittee members (Fig. 1). Study Group chairs are selected by the Subcommittee chairs and subsequently invite Study Group members to contribute their expertise relating to a particular virus taxon or discipline (such as virology, structural biology, bioinformatics, evolutionary biology or medicine). The ICTV members’ election procedures, tenure and duties are described in detail in the ICTV Statutes. The decisions of the ICTV EC and ICTV are made by majority voting. The ICTV facilitates the implementation of virus taxonomy by authorizing changes in response to submitted taxonomic proposals (TaxoProps). This process contrasts with the classification systems of animals and plants, in which the recognition and naming of species are traditionally based on valid publication and specimen deposition, with priority being given to the first description of a taxon. All members of the ICTV EC and Study Groups are volunteers and do not receive any financial remuneration for their work.

## ICTV taxonomy

The nature and origins of viruses have engaged virologists for decades [5, 6]. Viruses are biological entities that replicate and have co-evolved intimately with their cellular hosts for billions of years. The classification and naming of viruses have an essential role in clinical, agricultural, educational and regulatory fields.

**Fig. 2.**
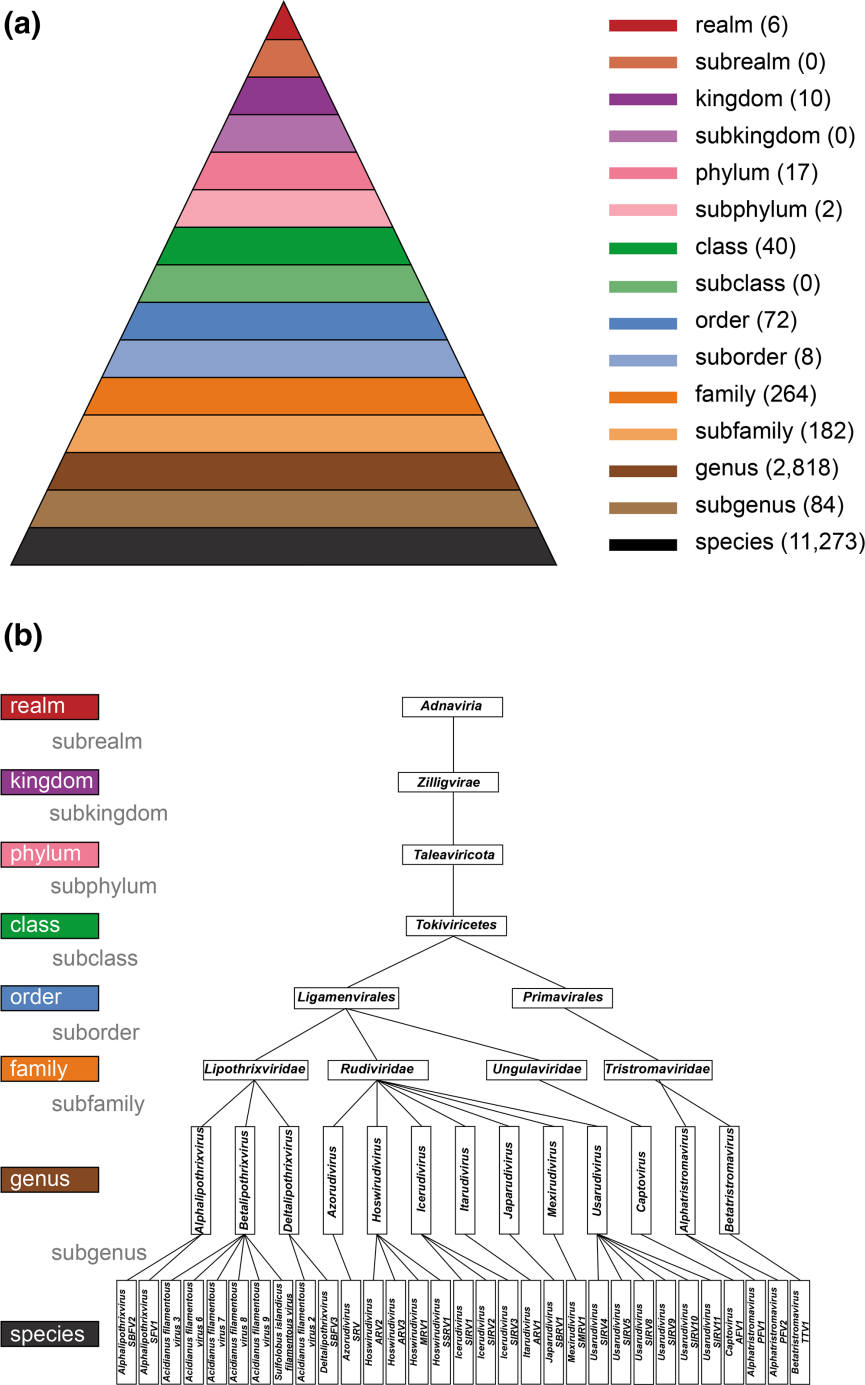
The ICTV taxonomic rank hierarchy. (a) The ICTV-authorized 15-rank hierarchal taxonomy of viruses as ratified in 2023. Taxonomic ranks are shown with an idealized distribution pattern of taxa; actual numbers of taxa are shown in brackets. When the ranks are described as a hierarchy, the species rank is often referred to as the lowest rank and the realm rank as the highest rank. When the ranks are used as phylogenetic terms, the realm rank may be described as basal, and the species rank as apical. (b) Taxonomic structure of the realm *Adnaviria* [45]. Species taxon names are as currently assigned, but will be revised to binomial format in 2023.

Viruses are classified into taxa that are placed into hierarchical ranks; the primary ranks are named realm, kingdom, phylum, class, order, family, genus and species [7]. Additionally, secondary ranks are available between the primary ranks (e.g. subrealm, subkingdom and subphylum), but there is no rank below that of species (Fig. 2a). This 15-rank taxonomy is designed to accommodate the entire spectrum of genetic diversity and evolutionary relationships in the virosphere and is reminiscent of the Linnaean taxonomic system for cellular organisms. The guiding principle for current virus taxonomy is that taxonomic assignments should be congruent with inferred virus evolutionary histories; i.e. taxa should be monophyletic [4], albeit with the caveat that virus genomes can have complex reticulate evolutionary histories. Phenotypic and ecological properties of viruses may be used to inform the boundaries between ranks, but all taxa should be monophyletic and their relationships based on evolutionary analysis of appropriate genomic characters. At present, 80 % of 11 273 virus species taxa are assigned to a family taxon and 94 % of species taxa have been assigned to one of the six currently established realms. Assignment to ranks other than genus and species is not mandatory.

Taxonomy is a dynamic science that develops as analytical methods improve and new data become available. For example, there are six realms, each of which is inferred to represent a unique monophyletic evolutionary origin of its constituent viruses. However, recent evidence suggests that the number of independent and ancient evolutionary origins of viruses may be larger [8], implying that revisions to the current taxonomy, especially at the highest ranks, are inevitable. A functioning virus taxonomy must also recognize and rectify incorrect classifications as new information becomes available. For example, from 1975 onwards the genus *Rubivirus* was included in the family *Togaviridae* along with members of the genus *Alphavirus*. However, rubiviruses (including rubella virus) differ considerably from alphaviruses such as Semliki Forest virus and chikungunya virus in their mode of transmission, virion structure and sequence relationships. Thus, in 2019, the genus was moved to a newly created family, *Matonaviridae*, which now includes both human and animal viruses [9].

The ICTV is charged with the development of a universal taxonomy that can be applied to all viruses irrespective of their hosts, whether they have been isolated and characterized phenotypically or if they have been identified only via sequences of nucleic acids in the metagenomic (or metatranscriptomic) analysis of viromes or environmental samples [10].

The term “universal” does not mean that all viruses must be classified and named using a single approach or placed within a single tree. Nor does it mean that the ranking of taxa for different virus lineages must necessarily represent equivalent degrees of genetic divergence. The problems of “ranking” in natural classification systems are well known [11] and they apply equally well to virus classification [12]. There is also no requirement for all taxonomic ranks to be used. For example, in the realm *Adnaviria*, sub-ranks are not used (Fig. 2b), and there are many other instances in which several primary and secondary ranks have not yet been assigned (Table 1).

**Table 1.** Examples of virus taxonomy

RANK	Taxonomy								
**realm**	*Adnaviria*	*Duplodnaviria*	*Monodnaviria*	*Riboviria*	*Riboviria*	*Ribozyviria*	*Varidnaviria*		
**kingdom**	*Zilligvirae*	*Heunggongvirae*	*Sangervirae*	*Orthornavirae*	*Orthornavirae*		*Bamfordvirae*		
**phylum**	*Taleaviricota*	*Uroviricota*	*Phixviricota*	*Lenarviricota*	*Pisuviricota*		*Preplasmiviricota*		
**class**	*Tokiviricetes*	*Caudoviricetes*	*Malgrandaviricetes*	*Leviviricetes*	*Pisoniviricetes*		*Polintoviricetes*	*Naldaviricetes*	
**order**	*Ligamenvirales*	*Crassvirales*	*Petitvirales*		*Nidovirales*		*Orthopolintovirales*	*Lefavirales*	
**suborder**					*Arnidovirineae*				
**family**	*Lipothrixviridae*	*Crevaviridae*	*Microviridae*		*Arteriviridae*	*Kolmioviridae*	*Adintoviridae*	*Baculoviridae*	*Ampullaviridae*
**subfamily**		*Coarsevirinae*	*Bullavirinae*		*Variarterivirinae*				
**genus**	*Alphalipothrixvirus*	*Junduvirus*	*Gequatrovirus*	*Hohglivirus*	*Betaarterivirus*	*Daazvirus*	*Alphadintovirus*	*Alphabaculovirus*	*Bottigliavirus*
**subgenus**					*Chibartevirus*				
**species**	*Alphalipothrixvirus* *SBFV2*	*Junduvirus* *communis*	*Gequatrovirus* *talmos*	*Hohglivirus* *simiicola*	*Betaarterivirus* *sheoin*	*Daazvirus* *cynopis*	*Alphadintovirus* *mayetiola*	*Alphabaculovirus* *ardigrammae*	*Bottigliavirus* *ABV*

**Fig. 3.**
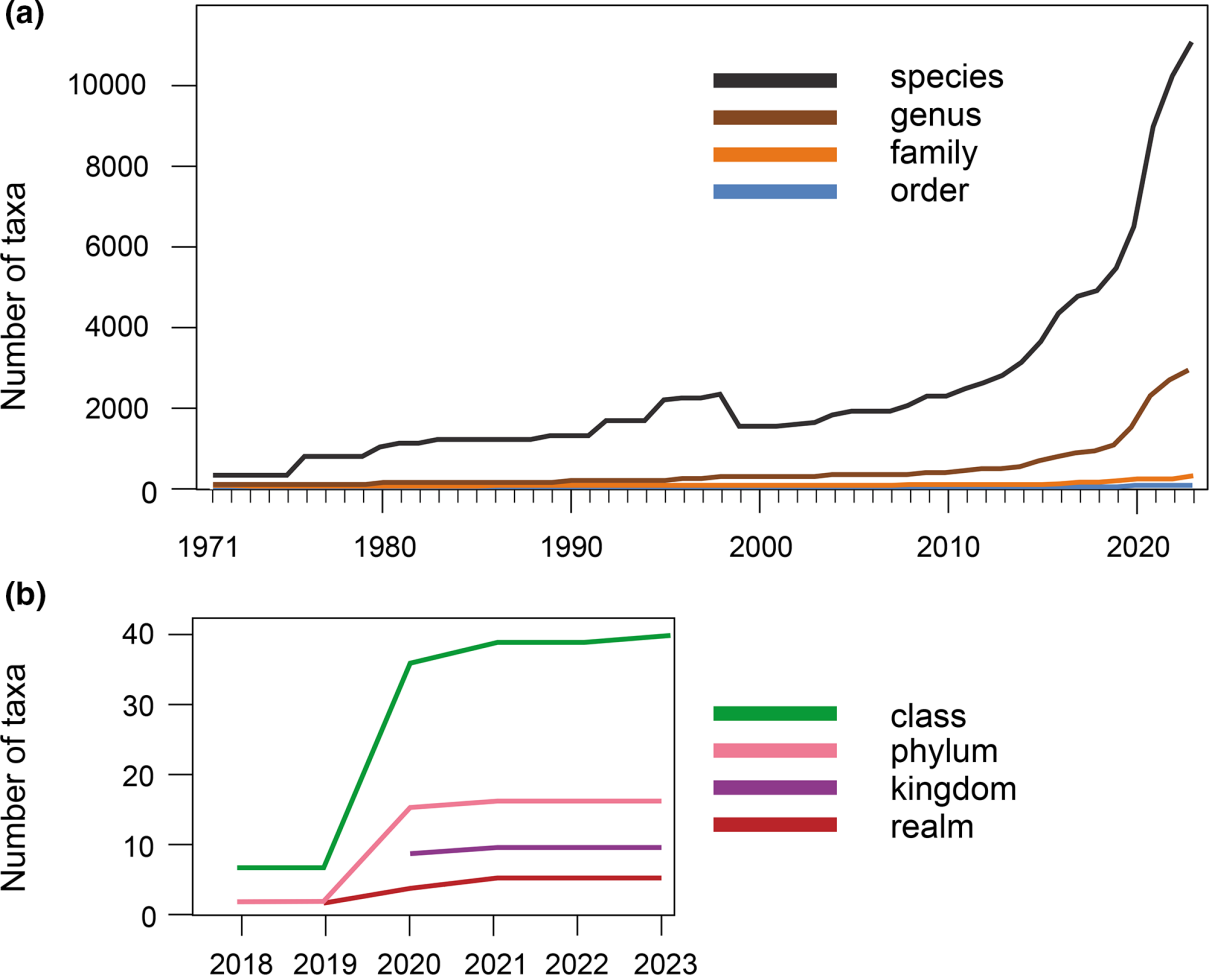
The number of taxa in primary ranks. (a) The number of taxa in the species, genus, family, and order ranks, 1971–2023. The drop in species taxa numbers in 1999 was due to some closely related viruses being consolidated into single species taxa at the same time as the systematic introduction of virus species taxa. (b) The number of taxa in the class, phylum, kingdom and realm ranks, 2018–2023. Note that the MSL in which these changes occurred generally has the previous calendar year as an identifier, indicating the year of the EC meeting at which the taxonomic changes were proposed.

A description of the many methods that can be used to infer the evolutionary histories of viruses and support classification decisions is beyond the scope of this article but has been summarized elsewhere [4]. These may focus on virus genomes, hallmark genes or virus-encoded proteins, and range from the simple calculation of pairwise nucleotide distances to the comparison and classification of virus protein structures [13, 14]. As conflicting evolutionary signals can be provided as a result of recombination, e.g. from the acquisition of genes from other viruses or hosts, different approaches have been developed to disentangle alternative evolutionary histories [15, 16]. The decision regarding which methods are used is left to the authors of taxonomic proposals, usually, but not always, the taxon-specific Study Groups that include experts with domain-specific knowledge. Again, this emphasizes the community-based origin of virus taxonomy. The current taxonomy is available as the Master Species List (MSL) at https://ictv.global/msl and can be searched using the taxonomy browser at https://ictv.global/taxonomy. The number of taxa in each of the primary taxonomic ranks from 1971 onwards is shown in Fig. 3.

## ICTV processes

### Taxonomic proposals (TaxoProps)

The process used to create or abolish taxa, to change taxon names, or to change the position of taxa in the taxonomic hierarchy begins with the submission of a TaxoProp to the ICTV. Although most proposals are submitted by taxon-specific Study Groups, which are tasked by the ICTV EC with the classification of viruses within specific families or other high-rank taxa, anyone can debate and submit a proposal for consideration. There are several websites that collate information on particular groups of viruses, including viruses that have yet to be classified (e.g. www.picornaviridae.com, sites.google.com/site/adenoseq). TaxoProps are submitted to the Subcommittee chair for the relevant group of viruses (https://ictv.global/sc); i.e. animal DNA viruses and retroviruses, animal dsRNA and (-)RNA viruses, animal (+)RNA viruses, archaeal viruses, bacterial viruses, fungal and protist viruses, and plant viruses (Fig. 1). Subcommittee chairs may propose revisions to prepare TaxoProps for consideration by the EC. If the taxa within the proposals span multiple hosts or virus groups, proposals may be submitted to multiple Subcommittee chairs. TaxoProps are evaluated once a year; the deadline for submission is typically in May/June, with the initial review at the EC meeting in July/August. Proposals that are not approved by the EC are either rejected or returned to the authors for amendment before reconsideration.

When a TaxoProp is approved by the EC, it will enter a ratification vote by the full ICTV membership (Fig. 1); this vote typically takes place early in the year following the EC meeting at which the proposal was accepted. If ratified, the now accepted taxonomic changes will be published online in the MSL and Virus Metadata Resource (VMR) (see below). At all stages of the process, each TaxoProp is posted publicly for comment (https://ictv.global/files/proposals/pending); ratified TaxoProps are archived at https://ictv.global/files/proposals/approved. TaxoProp templates and instructions can be downloaded from https://ictv.global/taxonomy/templates.

TaxoProps take the form of Microsoft Word documents summarizing the evidence for the proposed taxonomic changes or additions, and Excel spreadsheets itemizing any new taxa, as well as changes to existing taxonomic names or their place in the taxonomic hierarchy. TaxoProps must propose taxon names, provide supporting evidence for proposed taxonomic placements, and in the case of new species taxa, must provide nucleotide sequence information via GenBank accession numbers of an exemplar virus isolate. All TaxoProps must be based on the availability of at least one near-complete genome sequence of an exemplar virus. The ICTV does not classify viruses characterized only by partial genome sequences. Particular care must be taken with the accuracy of the Excel spreadsheets, because the process of correcting even an incorrectly spelled taxon name can be lengthy and may require submission of a corrected TaxoProp.

One of the most important elements of a TaxoProp is the evidence that supports the assignment of a group of viruses into a taxon and the placement of that taxon in a ranking hierarchy that is built upon the inclusion principle (see Glossary). Thus, a proposal to classify a group of viruses for the first time must be clear about the criteria used to assign viruses to a taxon at the species rank and assign species to higher-rank taxa. At a minimum, species taxa must be assigned to a genus and, optionally, other higher-rank taxa. TaxoProps normally include a phylogenetic analysis of relevant nucleotide or amino-acid sequences, or present alternative evidence justifying monophyly. If the viruses are to be classified in an existing taxonomy, the established assignment criteria must be used or a convincing scientific argument to change these criteria must be presented.

### Orthography

In virus taxonomy, the names of all taxa are written in italics. Names of taxa placed in ranks above species must end with a mandated suffix, as outlined in Table 2. After consultation [17], discussion by the EC, and a ratification vote, species taxon names are required to be written in a binomial format that is comprised of the genus name followed by a space and then a species epithet. Species epithets can be in any form as long as they are a single word made of letters from the Latin alphabet and numbers only. Species epithets may be Latinized [18]; guidance on the format of non-Latinized epithets is available at https://ictv.global/taxonomy/templates. The binomial format requirement is mandated for all new species taxon names and is being introduced retrospectively for existing names, with a deadline of the EC meeting of 2023.

**Table 2.** Mandated suffixes for virus taxon names

Rank	Suffix
realm	…*viria*
subrealm	…*vira*
kingdom	…*virae*
subkingdom	…*virites*
phylum	…*viricota*
subphylum	…*viricotina*
class	…*viricetes*
subclass	…*viricetidae*
order	…*virales*
suborder	…*virineae*
family	…*viridae*
subfamily	…*virinae*
genus	…*virus*
subgenus	…*virus*
species	not applicable

## ICTV resources

### Master Species List and Virus Metadata Resource

The ICTV maintains a list of all virus taxa and their history, called the Master Species List (MSL). The MSL is updated each year following the ratification vote and is available from the ICTV website (https://ictv.global/msl). A complementary list, called the Virus Metadata Resource (VMR), provides information about at least one virus assigned to each species taxon, including the virus name, an accession number for all or part of the virus genome, the nature of the virus genome (DNA or RNA, single- or double-stranded and positive- or negative-sense), and the natural host (plant/animal/fungi/protist/bacteria or archaea), if known. This means that, by searching the VMR, it is possible to link virus names to the corresponding species and higher taxa and to link virus species taxa with the genome sequence of an exemplar virus (https://ictv.global/help/vmr/find_species_name). The VMR is updated several times a year and is available as a downloadable spreadsheet (https://ictv.global/vmr).

The MSL and VMR also form the basis of the virus section of the National Center for Biotechnology Information (NCBI) Taxonomy Database [19]. However, implementation of the new taxonomy releases into the NCBI database is not automatic, and short delays are expected each year. Thus, the most recent MSL and VMR databases should be used to confirm virus taxonomic assignments.

### The ICTV website

The ICTV website provides access to the current taxonomy database in online and downloadable formats and maintains a complete history of virus taxa dating back to the first release in 1971 (https://ictv.global/taxonomy/history). The site also provides information about the ICTV, recent news on virus taxonomy and forums for general and taxonomic discussions.

### The ICTV Report and Profiles

The ICTV Report is published online as a continuously updated open-access publication (https://ictv.global/report). It strives to be a comprehensive description of all virus taxa and includes extensive information on virion structure, genome structure and replication, biology and phylogeny. Chapters typically refer to a particular virus family and are written by ICTV Study Groups and edited by members of the EC. In addition, summaries of the individual ICTV Report chapters are published in a special section of the *Journal of General Virology*, called “ICTV Virus Taxonomy Profiles” (http://www.microbiologyresearch.org/content/ictv-virus-taxonomy-profiles). These Profiles provide concise summaries of the classification, structure and properties of members of the virus taxa included in the Report chapter. The Profiles are indexed in PubMed (for example, [20, 21]) and can be used to cite the corresponding chapters of the ICTV Report, even when the content of the Report has been subsequently updated.

### Virology Division News

The Virology Division News (VDN) section of *Archives of Virology* is currently an important freely accessible vehicle for communicating ICTV news to virologists and others (https://www.springer.com/journal/705/updates/18970520). For example, previous VDN reviews and articles have encompassed the development of virus taxonomy, including official taxonomic changes [22], the substance of taxonomic proposals under consideration by the ICTV [23] and informal suggestions for discussion [17].

## Virus classification and nomenclature below the rank of species

### Classification

The remit of the ICTV only extends to the assignment, placement and naming of taxa at and above the species rank. No general guidance can be given about classification below the species rank because criteria differ across groups of viruses. For example, viruses assigned to the same species taxon may be grouped into different serotypes or genotypes based upon cross-neutralisation or genetic divergence of their nucleotide or encoded amino-acid sequences. Examples include the numerous (sero)types of enteroviruses within the species *Enterovirus A* (family *Picornaviridae*) or (geno)types of avian hepatitis E viruses (species *Avihepevirus magniiecur*, family *Hepeviridae*). Other viruses belonging to a single species taxon have been grouped using other biological characteristics, such as disease associations, host range or virion structure. In some cases, there may be a need to group viruses that are not congruent with evolutionary relationships, and these classifications may include viruses from several species taxa. This is often the case when the groupings have relevance for disease, bioregulation or biology. However, these alternative classifications cannot be regarded as virus taxonomies.

### Nomenclature

An issue separate from classification below the level of the species taxon is the names given to viruses as physical entities or the diseases they produce. As an example of the use of these different kinds of names, rubella virus (the virus name), the etiological agent of rubella or German measles (the disease names), is a member of the species *Rubivirus rubellae* (the species taxon name) in the family *Matonaviridae* (the family taxon name). Virus names are not regulated by the ICTV [1, 24] and are usually those used in the first description of the virus, either from a publication or a nucleotide accession entry. To help distinguish virus names from taxon names (especially the species taxon name), virus names should be written without italics. They should also be written without capital letters except for proper nouns that are virus name components. In addition, it is preferable for virus names to be distinct from virus species taxon names to help avoid category confusion, something that has been particularly widespread for plant and bacterial viruses. The confusion of virus and species taxon names should become less acute following the mandatory use of binomial virus species taxon names, but the advice for naming viruses is still valid. The use of place names or personal names as parts of virus names should be carefully considered before being adopted, as the chosen name may not be unique or the association with a virus may be unwelcome [25].

Another subsidiary issue concerns the naming of variants or strains of the same virus. There is no established convention for this within the field of virology and the ICTV has no remit in this matter. For some viruses, isolates are named according to a defined pattern. For example, for influenza A viruses (species *Alphainfluenzavirus influenzae*, family *Orthomyxoviridae*), the isolate designation form is type/host (if not human)/country/strain number/year (haemagglutinin/neuraminidase type)—giving “influenza A virus, isolate A/swine/Italy/81220/2017(H1N1)”. Such names may simplify the computational analysis of virus sequences, but they can also become cumbersome. For bacterial viruses, a common naming pattern takes the form “Acinetobacter phage Apostate” or the formulaic “Acinetobacter phage vB_AbaM_Apostate”, consisting of the virus host genus followed by “phage” and a unique phage name, which can take the form “vB” (virus of bacteria), “Aba” (abbreviated host genus and species name), “M” (virus morphotype, indicating myovirus in this case), followed by the phage common name or identifier which should be unique [26, 27].

## Conclusions

In the last few years, virus taxonomy has changed from a specialist niche to an interdisciplinary field that encompasses virology, bioinformatics, evolutionary biology and, occasionally, philosophical considerations on the meaning of taxonomy, particularly when inferring deeper, less compelling, evolutionary relationships [6, 28–33] or the nature of virus species and the associated principles of categorization [34–36]. This change of scope has largely been due to the introduction of high-throughput sequencing technology [37, 38], recognition of the true diversity of the virosphere [8, 39–41] and progress in computational phylogenetics [42–44]. The next decade of virus taxonomy will be very challenging and requires a continuing effort to unravel the genetic make-up and evolution of virus genomes, as these are shaped by biological and ecological mechanisms. The goal of this effort, which is supported by the ICTV, is to translate this knowledge into a taxonomy that helps us make more sense of the natural world.

## Glossary

Classification. The rational process of assigning viruses to variously defined groups.

Hallmark genes. A group of orthologous genes or gene sets, that are evolutionary conserved and used in the construction of a hierarchical taxonomy.

Inclusion principle. Viruses assigned to a lower taxon based on certain virus characteristics must also have the characteristics needed for classification into a higher-ranked taxon that includes the lower taxon.

Monophyletic. A monophyletic taxon is defined as one that includes a single most recent common ancestor of a group of viruses and all of its descendants.

Nomenclature. The assignment of names to taxa.

Phylogenetics (of viruses). The inference of evolutionary history and relationships among or within groups of viruses.

Polyphyletic. A polyphyletic taxon is defined as one that groups together the descendants of more than one most recent common ancestor.

Ranking. In biological classification, the relative level of a group of organisms (a taxon) in an ancestral or hereditary hierarchy.

Reassortment (or pseudorecombination). The exchange of one or more genetic segments that occurs in viruses with segmented genomes.

Recombination. The combining of genetic information between nucleic acid molecules from divergent genomes. Recombination (involving the homologous or non-homologous exchange of genetic material) occurs within a virus genome, between divergent co-infecting viruses or between virus and host (leading to gene acquisition).

Taxon. A monophyletic category that includes viruses sharing certain characteristics related by descent from a common ancestor. The commonly accepted correct plural of “taxon” is the fake Greek “taxa”.

Virome. The assemblage of viruses found associated with a particular ecosystem, organism or community of organisms.

Virosphere. The pool of viruses in all hosts and all environments on planet Earth.

Virion. Extracellular infectious virus particles that are capable of initiating a virus replication cycle.

Virus. A replicative non-cellular entity that is obliged to depend on a host cell for replication.

Virus taxonomy. The classification of viruses into taxa and taxon nomenclature.
